# SDQ: discriminative validity and diagnostic potential

**DOI:** 10.3389/fpsyg.2015.00811

**Published:** 2015-06-10

**Authors:** Thaysa B. F. Silva, Flávia L. Osório, Sonia R. Loureiro

**Affiliations:** Department of Neurosciences and Behavior, University of Sao PauloRibeirão Preto, Brazil

**Keywords:** behavioral problems, validity, children's psychopathology, psychometrics, mental health

## Abstract

The Strengths and Difficulties Questionnaire (SDQ) was designed to screen for behavioral problems in youths based on cutoff points that favor the instrument's diagnostic sensitivity. The present study aimed to analyze the discriminative validity of the SDQ to identify behavioral difficulties and prosocial resources in school-age children compared with the diagnostic data collected by the corresponding sections of the Development and Well-being Assessment (DAWBA). In addition, new cutoff points that value specificity were defined for the SDQ scales, exploring its diagnostic potential. This study was conducted in Brazil and assessed a community convenience sample that consisted of 120 children aged 6–12 years who were not under psychological/psychiatric treatment. The mothers of the participants also completed a sociodemographic questionnaire. Descriptive statistics were used to clinically characterize the sample. A ROC curve was used to assess the discriminant validity of the SDQ, and new cutoff points were established to maximize the instrument's specificity. The new cutoff points enabled a significant increase in specificity without a significant loss of sensitivity, which favors approaches based on measures of screening and diagnosis yet does not damage the instrument's screening capacity. The following increases were observed: 100% for the depressive disorder scale (cutoff point = 7), 95.1% for the generalized anxiety disorder scale (cutoff point = 7), 46.6% for the conduct disorder scale (cutoff point = 6), 19.2% for the hyperactive disorder scale (cutoff point = 8), and 27.6% for the antisocial personality disorder scale (cutoff point = 6). A cutoff point of 8 was applied to the prosocial behavior scale, which exhibited a 62.1% increase in specificity. The use of more specific cutoff points generated more accurate results and favored SDQ's use, particularly in contexts of care that require more precise and faster procedures for identification of problems.

## Introduction

Although rarely diagnosed, mental health problems are common in children and adolescents. According to epidemiological data, with reference to the diagnostic criteria of the Diagnostic and Statistical Manual of Mental Disorders IV (DSM-IV), the prevalence of mental disorders in children and adolescents in general-population samples is 10–15%. The most prevalent disorders are conduct (7.0%) and anxiety (5.2%) disorders (Fleitlich-Bilyk and Goodman, 2004; Goodman et al., 2004). In a study conducted with a community sample in Brazil, it was found that 10.8% of children had at least one psychiatric disorder according to the International Classification of Diseases 10 (ICD-10) or DSM-IV (Anselmi et al., 2010). In the United States, a study diagnosed 8% of children and adolescents from a community sample with depression or anxiety. Additionally, 5.4% had been diagnosed with behavioral problems (Ghandour et al., 2012). Regarding mental health problems among children and adolescents from clinical samples, a Brazilian study found a prevalence rate ranging from 7.8% (learning disabilities) to 28.7% (attention deficit and hyperactivity disorder and disruptive behavior disorder) (Delvan et al., 2010).

These mental health problems in childhood not only affect child development but also increase the risk of psychosocial disorders in adulthood (Ferrioli et al., 2007). According to country, there is a discrepancy between the prevalence of mental health problems and those treated during childhood and adolescence. This inadequate care contrasts starkly with the magnitude of the mental health problems and associated consequences observed in youths (Couto et al., 2008).

One reason for the low treatment demand is that children's mental health problems are often not identified or diagnosed. Thus, an increasing need exists for more precise information. Within this context, the need for assessments stands out because their application with regard to the screening and diagnosis of mental disorders might enable healthcare services to provide for their communities, establish bases for treatment, and formulate prevention programs (Fleitlich and Goodman, 2000; Mendes, 2009).

Several screening instruments are available, and the instruments that can detect problems early and quickly have received special attention, since the reducing of the impact and incidence of mental health problems need early detection (Stone et al., 2010). Numerous questionnaires have been formulated over recent decades to assess psychopathological indicators in youths. The questionnaires of Rutter and Achenbach (Rutter, 1967; Achenbach, 1991) stand out. Although these questionnaires are widely used, their length limits their use. Thus, Goodman (1997) developed the Strengths and Difficulties Questionnaire (SDQ) to satisfy the clinical need for a short, simple, and clinically useful questionnaire that is well accepted by respondents (Fleitlich et al., 2000).

The SDQ has been translated into more than 60 languages and is widely used across different cultures (Stone et al., 2010). Fleitlich and Goodman translated and adapted this instrument into Portuguese (Fleitlich and Goodman, 2001). A study had described the instrument's psychometric properties in Brazil and found satisfactory results relative to the instrument's discriminative validity, reliability, and internal consistency (Woerner et al., 2004a). Two different methods were used to evaluate the validity of the SDQ. One method was to compare the means of scales of a community sample in relation to the means of a clinical sample of patients diagnosed with psychiatric disorders. The results showed significant differences between the total score of the community sample of the clinical sample versions of parents and teachers. Another method of assessing the validity of the SDQ was the comparison between the assessment of mental health problems through the SDQ and the assessment of psychiatric disorders using a diagnostic measure, DAWBA. For this purpose were randomly selected 41 community participants with SDQ indicative of the presence of difficulty, and 56% had some confirmation diagnosis by DAWBA. Were also selected 40 community participants with SDQ indicating no difficulty, and only 15% had a diagnosis confirmation of DSM-IV in the evaluation by DAWBA.

Like the studies conducted in Brazil, the studies conducted abroad that have assessed the SDQ's psychometric properties emphasize the appropriate use of this instrument for screening as opposed to diagnosis. The cutoff points are associated with the sensitivity (Se) and specificity (Sp) of instruments. Sensitivity is related to the proportion of positive cases correctly identified by the instrument, and the specificity is related to the proportion of negative cases evaluated correctly. Therefore, the cutoff points must be evaluated based on the particular purpose of each instrument. Consequently, the cutoff points consistently favor the Se of the instruments used for screening and the Sp of those instruments used for diagnostic purposes.

The prosocial behavior scale assesses resources rather than problems. Thus, its score is not included in the total difficulty score because a lack of prosocial behavior problems is conceptually different from the presence of psychological difficulties (Goodman, 1997). Prosocial resources might be related to social skills or competences. Socially competent children are those who exhibit adaptive behaviors relative to their same-age peers (Luiz et al., 2010).

The original study, developed by Fleitlich et al., suggested the cutoff points attributed to the various SDQ scales for the Brazilian population: 14 for the total difficulty score, 4 for the emotional symptoms scale, 3 for the conduct problems scale, 6 for the hyperactivity/inattention scale, 3 for the peer relationship problems scale, and 6 for the prosocial behavior scale (Fleitlich et al., 2000). These cutoff points are used to screen for psychosocial difficulties exhibited by children. Thus, they favor Se over Sp. The enhancement of Se over Sp was also identified by studies conducted in other countries. A Norwegian study (Hysing et al., 2007) found an 81.8% Se and a 69% Sp for the parent version of the SDQ when the 90th percentile was established as the cutoff point for all scales.

Studies on the parent version of the SDQ have been conducted in several countries, such as Japan (Matsuishi et al., 2008), Germany (Woerner et al., 2004b), United States (Bourdon et al., 2005), France (Shojaei et al., 2009), and China (Du et al., 2008). In these studies, it was noted that the cutoff points were selected to favor the questionnaire's Se over its Sp. Regarding the Brazilian version of the SDQ, the cutoff points differ from those of these countries in certain scales but are generally similar. Furthermore, as noted in other countries, the cutoff points applied to the Brazilian population also favor the instrument's Se.

Based on the properties of the SDQ, it is reasonable to examine whether this screening instrument could be successfully used as a diagnostic tool. If so, then the cutoff points should favor the instruments' Sp to avoid the occurrence of false-negative results. A literature review did not locate any study that pursued such an aim in a Brazilian population (Saur and Loureiro, 2012).

The present study analyzed the discriminative validity of the SDQ to identify behavioral difficulties and prosocial resources in school-age children compared with the diagnostic data collected by the corresponding sections of the Development and Well-being Assessment (DAWBA). New cutoff points were defined for the SDQ scales that valued the Sp in order to improve its utility as a diagnostic measure whilst retaining its important properties as a screening tool. Psychometric studies have always used the SDQ as a screening instrument. No study has sought to establish cutoff points that value the specificity to adapt the screening instrument for use as a diagnostic measure. The improvement of the SDQ screening process could increase the value of its applicability, which would present a significant clinical advantage by decreasing the evaluation time, remove the need to apply other instruments, and facilitate the implementation of priority referrals for care in children's mental health. The hypothesis of this study is that it will be possible to define empirically derived cutoff points for subscales of the SDQ which would value specificity, thus improving its diagnostic potential, without impairing its capacity as a screening instrument.

## Materials and methods

The present study employed a cross-sectional design in which the groups were compared using assessment measures. The children's mothers were personally recruited in the primary care context, in a basic health unit of Uberaba-MG, Brazil, when they were seeking care for themselves, not psychological or psychiatric. The mothers completed the screening and diagnostic instruments.

### Selection of participants

This study used a convenience community sample that consisted of 120 Brazilian children. The inclusion criteria were as follows: both genders, aged 6–12 years, residing with the biological mother, no apparent physical or sensory disabilities, and not receiving psychological or psychiatric treatment at the time of the study.

The children belonged to a population of mothers who received routine primary care during visits at general medicine and gynecology services. A total of 608 women visited these services over four consecutive months. A total of 251 were mothers of children who met the inclusion criteria. Of these mothers, 43 refused to participate because of a lack of available time. Of the remaining 208 mothers with eligible children, 177 allowed their children to receive a behavioral assessment and 31 mothers did not complete the assessment at their child's diagnostic interview. For convenience, the first 120 mothers who agreed to participate and completed their children's behavioral and diagnostic assessments were selected.

### Ethics statement

The ethics committee of the University of Uberaba approved this study (CAAE: 0030.0.227.000-06; CAAE: 0002.0.227.000-10), and the children's mothers signed informed consent forms for their participation. The children's assent was also considered. The consent terms were presented to the mothers verbally, in writing, and by reading together with researcher responsible for the assessment. The mothers were informed of the study objectives, the absence of loss or damage arising from participation, and the commitment to confidentiality with regard to the information obtained in the survey. It was made clear that participation was voluntary and emphasized that the mothers could withdraw at any time during the study without any negative consequences to themselves or their children.

### Instruments

The following instruments were used for data collection:

1. SDQ. The SDQ is a 25-item, open-access document that is used to screen youths for behavioral problems. The respondent is asked to reply based on the behavior of the study child over the past 6 months. Five items refer to prosocial skills, and 20 refer to difficulties. These items are divided into five scales: emotional symptoms, conduct problems, hyperactivity/inattention, peer relationship problems, and prosocial behaviors. Each scale consists of five items answered on a scale of “not true,” “somewhat true,” or “certainly true.” The total difficulty score is calculated by adding the results of the scales (excluding the prosocial behavior scale). From the total difficulties score, the SDQ enables researchers to classify subjects as normal, borderline, or abnormal, based on cutoff points. The values proposed by the original study for a Brazilian population for the inclusion of children in each classification are as follows: 0–13 for the normal category, 14–16 for the borderline category, and 17–40 for the abnormal category (Fleitlich et al., 2000). Children can be classified into these categories on each scale of the instrument, and the absolute values for this classification vary among the individual scales. The parent version of this scale, which was translated and validated for Portuguese by Fleitlich and Goodman (2001), was used.

In Brazil, SDQ psychometric properties of reliability and discriminative validity were assessed and satisfactorily attested (Woerner et al., 2004a). To verify the reliability of the instrument, the test-retest method and the calculation of Cronbach's alpha were used, and significant values were reported. In this study, the test-retest reliability (20 days) ranged from 0.77 to 0.79.

(2) DAWBA. This instrument was designed to generate ICD-10 and DSM-IV diagnoses of mental disorders among 5–17-year-olds (Goodman et al., 2000). The parent version of this instrument was used in the present study. The version that was translated and adapted for Brazil by Fleitlich-Bilyk and Goodman was used (Fleitlich-Bilyk and Goodman, 2004). For the Brazilian adaptation, the DAWBA was translated into Portuguese and back-translated into English to check the reliability and discriminative validity, finding appropriate indicators (Fleitlich-Bilyk and Goodman, 2004). In this study, inter-rater reliability assessed by the kappa coefficient was 0.93 for the disorders in general.

For the purposes of this study, the sections of the DAWBA corresponding to the SDQ scales were applied, relative to the diagnostic assessments of depression (Depression), anxiety (Generalized Anxiety), attention and hyperactivity (Attention and Activity), interpersonal relationships (Friendship Questionnaire), difficult behaviors (Awkward and Troublesome Behavior), and social skills (Aptitudes Scale). DAWBA was used as the gold standard for the comparison with the SDQ scales, to analyze its discriminative validity to identify behavioral difficulties and prosocial resources in school-age children.

(3) Complementary Questionnaire. This survey was designed to collect the sociodemographic data of the children, such as age, schooling, presence of disabilities or chronic diseases, current and previous treatments, and medication of continuous use; and data of their families, such as socioeconomic status according to the Brazilian Association of Market Research (ABIPEME), family composition and variables related to the family environment, life events and social support.

### Data collection

Three psychologists with considerable clinical experience who were trained to apply the instruments conducted individual interviews with the mothers of the children who met the inclusion criteria at the homes of the mothers. The interviews were performed in person based on each instrument's specifications in a single session that lasted approximately 40 min.

### Data analyses

The clinical characteristics of the sample were analyzed using descriptive statistics. To analyze the discriminative ability of the SDQ, a receiver operating characteristic (ROC) curve was used to establish the cutoff points to diagnose depression, generalized anxiety, conduct disorder, ADHD, and antisocial personality disorder (i.e., to distinguish cases from non-cases), as well as the cutoff points for the social resources assessed by the interpersonal relationships scale of DAWBA.

The Se and Sp values, as well as the miscalculation rate (MCR), the positive predictive value (PPV), and the negative predictive value (NPV), were calculated. Se is calculated by the proportion of positive cases correctly identified by the instrument, and Sp is calculated by the proportion of negative cases evaluated correctly (Menezes and Nascimento, 2000). The MCR is the proportion of subjects incorrectly classified as positive or negative. The PPV indicates the probability that cases detected as positive are actually positive, and the NPV refers to the probability that cases detected as negative are actually negative (Menezes and Nascimento, 2000).

The cutoff points were selected so that there was a gain of Sp over Se. Posteriorly, the cutoff points were associated with psychiatric disorders diagnosed by the DAWBA: (a) for mood/anxiety disorders, cutoff points were chosen that represented an increase of at least 90% of the Sp and a decrease of approximately 50% of the MCR; (b) for behavioral disorders (conduct, hyperactivity and antisocial), the criteria were an increase of approximately 20% of the Sp and a 15% decrease in the MCR; (c) for the prosocial behavior scale, the increase in the Sp with the smallest MCR value was prioritized.

The statistical analysis was performed using SPSS (Statistical Package for Social Sciences), 16.0 version.

## Results

### Sample sociodemographic characteristics

A total of 120 children (average age = 9.6 years, standard deviation (SD) = 1.7 years, range = 6 years, 2 months old-12 years, 11 months old) were assessed. All participants attended elementary school, whereby 50% ranged from preschool to third grade and 50% ranged from fourth to sixth grade. The gender distribution was even with 50% boys and 50% girls. All participants belonged to the lower and lower-middle socioeconomic classes, and most belonged to the lower class. Most mothers were married or had stable relationships (70.8%), and 29.2% were single parents.

### SDQ discriminative validity

Based on the SDQ cutoff points for each scale suggested by the original study (Mendes, 2009), the DAWBA gold standard confirmed diagnoses of 22.9% of depressive disorder cases, 29.6% of generalized anxiety disorder cases, 17% of conduct disorder cases, 58.7% of ADHD cases, 18.2% of antisocial personality disorder cases, and 55.6% of difficulties with social skills cases.

Given that the cutoff point of the SDQ to screen for emotional symptoms is 4 (Fleitlich et al., 2000), Table 1 describes the Se, Sp, MCR, PPV, and NPV relative to the other SDQ cutoff points for emotional symptoms using the DAWBA diagnoses of depressive disorder, generalized anxiety, and depressive with generalized anxiety disorder as parameters.

**Table 1:** **Psychometric indicators of the cutoff points for the SDQ emotional symptoms scale**.

**DAWBA gold standard**	**SDQ emotional symptoms: cutoff points**	**Se**	**Sp**	**MCR**	**PPV**	**NPV**
Depressive disorder	2	1.00	0.11	0.75	0.17	1.00
	3	0.95	0.27	0.63	0.20	0.96
	4	0.95	0.38	0.53	0.22	0.97
	5	0.79	0.59	0.38	0.27	0.94
	6	0.74	0.68	0.31	0.30	0.93
	**7**	**0.68**	**0.76**	**0.25**	**0.35**	**0.93**
	8	0.58	0.85	0.19	0.42	0.91
	9	0.32	0.98	0.13	0.75	0.88
	10	0.16	0.98	0.15	0.60	0.86
Generalized anxiety disorder	2	1.00	0.11	0.71	0.22	1.00
	3	1.00	0.29	0.57	0.26	1.00
	4	1.00	0.41	0.48	0.30	1.00
	5	0.88	0.64	0.32	0.38	0.95
	6	0.83	0.73	0.25	0.43	0.95
	**7**	**0.75**	**0.80**	**0.21**	**0.49**	**0.93**
	8	0.63	0.89	0.17	0.58	0.90
	9	0.33	1.00	0.13	1.00	0.86
	10	0.21	1.00	0.16	1.00	0.83
Depressive and generalized anxiety disorders	2	1.00	0.12	0.66	0.26	1.00
	3	0.97	0.30	0.53	0.32	0.96
	4	0.97	0.42	0.44	0.36	0.97
	5	0.77	0.63	0.33	0.41	0.89
	6	0.73	0.73	0.27	0.48	0.89
	**7**	**0.67**	**0.81**	**0.23**	**0.54**	**0.88**
	8	0.57	0.90	0.18	0.65	0.86
	9	0.27	1.00	0.18	1.00	0.80
	10	0.17	1.00	0.21	1.00	0.78

*Se, Sensitivity; Sp, Specificity; MCR, Misclassification rate; PPV, Positive predictive value; NPV, Negative predictive value; Bold values, Suggested cutoff point*.

Based on the DAWBA diagnosis of depressive disorder, compared with the cutoff point of 4, a cutoff point of 7 achieved the best balance between Se and Sp, favoring the latter with a significant increase of 100% while decreasing the MCR by 52.8%. For this cutoff point, the area under the curve (AUC) value was 0.80 (*p* < 0.0001; *IC* = 0.68–0.91).

With respect to the SDQ screen for generalized anxiety disorder, the Se and NPV scores achieved the maximum value with cutoff points of 2, 3, and 4. Nevertheless, a cutoff point of 7 was associated with a 25% reduction in Se, a 95.1% increase in Sp, and a 56.3% decrease in the MCR compared with the previously recommended cutoff point of 4. The AUC value was 0.87 (*p* < 0.0001; *IC* = 0.80–0.95).

Finally, a cutoff point of 7 for the SDQ to detect at least one disorder (i.e., depression and generalized anxiety) compared with the cutoff point of 4 increased the Sp by 92.9% and decreased the MCR by 47.7%.

Therefore, as in the previous study (Fleitlich et al., 2000), a cutoff point of 4 favored the instrument's Se relative to depressive and generalized anxiety disorders. Thus, a cutoff point of 7 was more appropriate when Sp was favored in the screening of the investigated disorders, either individually or as a whole.

Concerning the remainder of the SDQ behavioral difficulty scales, the original study (Fleitlich et al., 2000) suggested a cutoff point of 3 for the conduct problems and peer relationship problems scales as well as a cutoff point of 6 for the hyperactivity/inattention scale. Table 2 describes the Se, Sp, MCR, PPV, and NPV scores relative to the various cutoff points for these SDQ scales using the corresponding DAWBA diagnoses of conduct disorder, ADHD, and antisocial personality disorder.

**Table 2:** **Psychometric indicators of the cutoff points for the SDQ conduct disorder, hyperactivity/inattention, and peer relationship problems scales (To be continued)**.

**DAWBA gold standard**	**SDQ: cutoff points**	**Se**	**Sp**	**MCR**	**PPV**	**NPV**
Conduct disorder	**CONDUCT PROBLEMS**					
	2	1.00	0.42	0.53	0.14	1.00
	3	0.90	0.58	0.39	0.16	0.98
	4	0.90	0.68	0.30	0.20	0.99
	5	0.70	0.83	0.18	0.27	0.97
	**6**	**0.70**	**0.85**	**0.16**	**0.30**	**0.97**
	7	0.30	0.94	0.12	0.30	0.94
	8	0.20	0.95	0.11	0.29	0.93
	9	0.10	0.98	0.09	0.33	0.92
	10	0.10	0.99	0.08	0.50	0.92
Attention deficit hyperactivity disorder	**HYPERACTIVITY/INATTENTION**					
	2	0.94	0.31	0.52	0.34	0.93
	3	0.94	0.49	0.38	0.41	0.96
	4	0.94	0.57	0.33	0.46	0.96
	5	0.88	0.72	0.23	0.55	0.94
	6	0.76	0.78	0.23	0.57	0.89
	7	0.45	0.89	0.23	0.60	0.81
	**8**	**0.45**	**0.93**	**0.20**	**0.71**	**0.82**
	9	0.33	0.99	0.19	0.92	0.80
	10	0.27	0.99	0.21	0.90	0.78
Antisocial personality disorder	**PEER RELATIONSHIP PROBLEMS**					
	2	1.00	0.50	0.48	0.11	1.00
	3	0.86	0.76	0.23	0.18	0.99
	4	0.86	0.83	0.17	0.24	0.99
	5	0.86	0.95	0.06	0.05	0.99
	**6**	**0.86**	**0.97**	**0.03**	**0.67**	**0.99**
	7	0.71	0.99	0.03	0.83	0.98
	8	0.29	1.00	0.04	1.00	0.96
	9	0.14	1.00	0.05	1.00	0.95

*Se, Sensitivity; Sp, Specificity; MCR, Misclassification rate; PPV, Positive predictive value; NPV, Negative predictive value; Bold values, Suggested cutoff point*.

Regarding the conduct disorder scale, a cutoff point of 6 compared with the previously recommended (Fleitlich et al., 2000) cutoff point of 3 was associated with a 22.2% reduction in Se but a 46.6% increase in Sp and a 59% decrease in the MCR. For this cutoff point, the AUC value was 0.85 (*p* < 0.0001; *IC* = 0.75-0.95). For ADHD detection, a cutoff point of 8 exhibited the highest Sp with a 19.2% increase in its value and displayed the smallest reduction in Se compared with the cutoff point of 6. The AUC value was 0.84 (*p* < 0.0001; *IC* = 0.75-0.92). Finally, a cutoff point of 6 for the SDQ regarding antisocial personality compared with the cutoff point of 3 exhibited the same Se value but a 27.6% increase in Sp and an 87% decrease in the MCR. For this cutoff point, the AUC value was 0.94 (*p* < 0.0001; *IC* = 0.84–1.04).

Therefore, the previously suggested (Fleitlich et al., 2000) cutoff points did not favor Sp with respect to the detection of conduct disorder, ADHD, and antisocial personality disorder. The most appropriate cutoff point to increase the diagnostic value of the instrument relative to conduct and antisocial personality disorders was 6, and 8 was the best indicator for the ADHD scale.

In the original study (Fleitlich et al., 2000), a cutoff point of 6 was suggested for the SDQ prosocial behavior scale. Unlike the other scales in which higher scores denote greater likelihoods of difficulties, a score of 6 or greater indicates the presence of resources for prosocial behavior. Table 3 describes the Se, Sp, MCR, PPV, and NPV scores that correspond to various cutoff points for the SDQ prosocial behavior scale based on the DABWA assessment of social skills.

**Table 3:** **Psychometric indicators of the various cutoff points for the SDQ prosocial behavior scale**.

**DAWBA gold standard**	**SDQ prosocial behavior: cutoff points**	**Se**	**Sp**	**MCR**	**PPV**	**NPV**
Social skills	2	1	0.03	0.28	0.72	1
	3	0.99	0.06	0.28	0.73	0.67
	4	0.99	0.09	0.27	0.73	0.75
	5	0.94	0.18	0.28	0.74	0.55
	6	0.91	0.29	0.27	0.76	0.56
	7	0.87	0.32	0.28	0.77	0.50
	**8**	**0.85**	**0.47**	**0.26**	**0.80**	**0.55**
	9	0.63	0.76	0.33	0.87	0.45
	10	0.47	0.79	0.44	0.85	0.37

*Se, Sensitivity; Sp, Specificity; MCR, Misclassification rate; PPV, Positive predictive value; NPV, Negative predictive value; Bold values, Suggested cutoff point*.

A cutoff point of 8 achieved the best Sp value, with an increase of 62.1%, in relation to the cutoff point of 6 but with the smallest MCR value. For this cutoff point, the AUC value was 0.72 (*p* < 0.0001; *IC* = 0.62-0.83).

## Discussion

Regarding the psychometric evaluation of the SDQ, it was expected that there would be cutoff points that could enhance the Sp of the instrument, thus exploring its diagnostic potential without harming its potential for screening. This hypothesis was confirmed. In addition, it was found that there are cutoff points that value the Sp without a significant loss of Se for the different SDQ scales.

The Se of a screening instrument is crucial (Goodman et al., 2004). In this regard, with the cutoff points proposed by the original study (Fleitlich et al., 2000), a pattern of recovery of the instrument's Se was identified. A similar pattern was found among studies conducted across several countries (Woerner et al., 2004b; Bourdon et al., 2005; Hysing et al., 2007; Du et al., 2008; Matsuishi et al., 2008; Shojaei et al., 2009), in which the SDQ was used to screen children for behavioral difficulties. Thus, the instrument's Se was favored. These studies, considering their aim of evaluating the SDQ as a screening measure, also established good indicators of the instrument's validity. However, the present study sought to improve diagnostic potential of SDQ for children's difficulties, by prioritizing the instrument's Sp.

When the cutoff points suggested by the previous study (Fleitlich et al., 2000) were applied to the present sample, the Sp of the various scales ranged from 0.29 to 0.78, which resulted in a high MCR. A high rate of children was found who had scores indicating the presence of the difficulties when assessed by the SDQ but without a diagnosis confirmed by the DAWBA. To explore the diagnostic potential of the SDQ, the cutoff points needed to be more specific. Therefore, other cutoff points were tested that increased the accuracy of screening for various childhood mental disorders more adequately with regard to each individual scale because their Sp was favored without significantly reducing their Se. The psychometric study data demonstrated that the most appropriate cutoff points (having as a basis the purpose of enhancing specificity) were as follows: 7 for the emotional symptoms scale, 6 for the conduct problems and peer relationships scales, 8 for the hyperactivity scale, and 8 for the prosocial behavior scale. When the suggested cutoff points were used in the current study, we observed an increase in Sp from 19.2 to 100% in different scales without a significant loss of Se values.

Several studies conducted across various countries have suggested new cutoff points for the SDQ scales based on the assessed population, also suggesting new norms to standardize the SDQ. Nevertheless, several of these new cutoff points differ from those suggested in the present study. Differences across cultures and populations as well as the differing aims of the various studies might explain this discrepancy. As a rule, the studies conducted outside Brazil assessed the screening potential of the SDQ and therefore did not prioritize its Sp. (In this study, Sp was prioritized.) Comparisons with other studies also indicate the need to adjust the cutoff points based on the sociocultural idiosyncrasies of the different populations and in particular the clinical assessments of children because of their developing characteristics.

Adopting the new cutoff point for each SDQ scale for the present sample, a significant decrease in the MCR was noted. This characteristic, coupled with the significant increase in Sp, might enhance the instrument's ability to avoid false-negative results, thereby increasing its precision with regard to the early detection of mental disorders in youths.

Regarding the MCR, it was noted that the highest values (even when the cutoff points were set) were related to the prosocial behavior and emotional symptoms scales. The lowest values were related to the peer relationships and conduct problems scales. A high MCR in the emotional symptom scale can be explained by the type of evaluation required because internalizing symptoms are not always recognized as interpersonal and behavior problems, which are more directly expressed in the environment.

The SDQ prosocial scale is the only scale that does not assess difficulties in childhood. Rather, it assesses children's resources. Furthermore, higher scores indicate more resources. In this scale, it was observed that with the use of higher cutoff points the MCR increased. It was expected to decrease because the Sp was favored. One possible explanation is that the assessment of the prosocial behavior by the SDQ differs from the evaluation of social skills by the DAWBA. In the SDQ, prosocial behavior is associated with a child's ability to relate well with peers, favoring actions that benefit the individuals with whom they live. This ability does not necessarily include the ability to cope and overcome conflicts and adversities in these relationships. However, the DAWBA, on its scale of social skills, evaluates these capabilities more comprehensively, including in its assessment the child's ability to address difficult situations, to be sufficiently flexible to cope with stressful or embarrassing situations, to know how to lose, and to recognize what is missed.

The new cutoff points enabled a significant increase in Sp without a significant loss of Se, which favors the approach of screening and diagnostic measures. Analyzing other studies, it was observed that there was a maintenance of Se (its level remained comparable with other studies) but an increase in Sp, which is the major contribution of this study (Woerner et al., 2004b; Bourdon et al., 2005; Hysing et al., 2007; Du et al., 2008; Matsuishi et al., 2008; Shojaei et al., 2009). The proposition of new cutoff points extended the study of the SDQ's discriminative validity. No other study has aimed to verify this instrument's diagnostic potential.

The use of the suggested cutoff points to unite the screening and diagnosis measures presents a significant clinical contribution. The SDQ is an instrument that is simple and fast to apply, and with the adaptation of its measures as a diagnostic instrument, it minimizes the need to use other tools, which are more complex and require application by specialized professionals. Thus, combined with the SDQ's high screening process accuracy, the instrument's use becomes even more appropriate, particularly in contexts of care that require more precise and faster procedures to identify problems, such as the primary care context, and when seeking to identify among the children using health services those individuals who primarily require mental health prevention and intervention measures.

One limitation of the present study is the use of a convenience sample that consisted of a small number of volunteers who were homogeneous and specific with respect to their socioeconomic and cultural levels and their stage of development. This sample hinders the generalization of the results. In addition, the present study did not use a clinical sample, and eligible children were identified via their mothers. Thus, to confirm the SDQ's diagnostic potential, future studies should target a defined clinical sample of children. Further studies should also be performed on children at different stages of development. Moreover, similar studies should be conducted to establish the discriminative validity and the diagnostic potential of the SDQ versions for teachers and children older than 11 years in Brazil.

### Conflict of interest statement

The authors declare that the research was conducted in the absence of any commercial or financial relationships that could be construed as a potential conflict of interest.
